# Establishment and Characterization of Three Human Ocular Adnexal Sebaceous Carcinoma Cell Lines

**DOI:** 10.3390/ijms251810183

**Published:** 2024-09-23

**Authors:** Su-Chan Lee, Cornelia Peterson, Kaixuan Wang, Lujain Alaali, James Eshleman, Nicholas R. Mahoney, Emily Li, Charles G. Eberhart, Ashley A. Campbell

**Affiliations:** 1 Department of Pathology, The Johns Hopkins University School of Medicine, Baltimore, MD 21205, USA; suchanlee80@gmail.com (S.-C.L.); lujain.alaali@gmail.com (L.A.); jeshlem@jhmi.edu (J.E.); 2 Department of Comparative Pathobiology, Tufts University Cummings School of Veterinary Medicine, North Grafton, MA 01536, USA; cornelia.peterson@tufts.edu; 3 Department of Oncology, The Johns Hopkins University School of Medicine, Baltimore, MD 21205, USA; kwang102@jhu.edu; 4 Department of Ophthalmology, The Johns Hopkins University School of Medicine, Baltimore, MD 21205, USA; nick.mahoney@jhmi.edu (N.R.M.); eli20@jhmi.edu (E.L.)

**Keywords:** sebaceous carcinoma, eyelid cancer, cell line, mitomycin C, next-generation sequencing

## Abstract

Ocular adnexal sebaceous carcinoma (SebCA) represents one of the most clinically problematic periocular tumors, often requiring aggressive surgical resection. The pathobiology of this tumor remains poorly understood, and few models exist that are suitable for preclinical testing. The aim of this study was to establish new cell lines to serve as models for pathobiological and drug testing. With patient consent, freshly resected tumor tissue was cultured using conditional reprogramming cell conditions. Standard techniques were used to characterize the cell lines in terms of overall growth, clonogenicity, apoptosis, and differentiation in vitro. Additional analyses including Western blotting, short tandem repeat (STR) profiling, and next-generation sequencing (NGS) were performed. Drug screening using mitomycin-C (MMC), 5-fluorouricil (5-FU), and 6-Diazo-5-oxo-L-norleucine (DON) were performed. JHH-SebCA01, JHH-SebCA02, and JHH-SebCA03 cell lines were established from two women and one man undergoing surgical resection of eyelid tumors. At passage 15, they each showed a doubling time of two to three days, and all could form colonies in anchorage-dependent conditions, but not in soft agar. The cells contained cytoplasmic vacuoles consistent with sebaceous differentiation, and adipophilin protein was present in all three lines. STR profiling confirmed that all lines were derived from their respective patients. NGS of the primary tumors and their matched cell lines identified numerous shared mutations, including alterations similar to those previously described in SebCA. Treatment with MMC or 5-FU resulted in dose-dependent growth inhibition and the induction of both apoptosis and differentiation. MYC protein was abundant in all three lines, and the glutamine metabolism inhibitor DON, previously shown to target high MYC tumors, slowed the growth of all our SebCA models. Ocular adnexal SebCA cell lines can be established using conditional reprogramming cell conditions, and our three new models are useful for testing therapies and interrogating the functional role of MYC and other possible molecular drivers. Current topical chemotherapies promote both apoptosis and differentiation in SebCA cells, and these tumors appear sensitive to inhibition or MYC-associated metabolic changes.

## 1. Introduction

Sebaceous carcinomas (SebCA) of the ocular adnexa most frequently arise from the Meibomian glands, commonly exhibit aggressive phenotypes, and complicate efforts for complete surgical resection, resulting in local invasion, tumor recurrence, and distant metastases [1,2,3,4,5,6]. SebCAs are rare, yet comprise approximately 5% of malignant epithelial eyelid tumors, with an incidence of 5 per 100,000 persons [7,8,9,10]. Several studies have identified mutations in tumor suppressor complexes and have correlated tumorigenesis with malignant transformation by high-risk human papillomavirus (HPV) [11,12,13,14,15,16,17,18,19]. Other investigators have associated ocular adnexal SebCA with concurrent visceral malignancies in patients with Muir–Torre Syndrome, characterized by microsatellite instability and autosomal dominant loss-of-function mutations in genes encoding mismatch repair proteins, although this is more common in extraocular SebCA [20,21,22,23,24,25,26]. Further, we have demonstrated an increased copy number at the *MYC* locus as well as high MYC protein levels in a subset of ocular adnexal SebCA [27]. However, the low incidence of ocular adnexal SebCA in the US and the generally fragmented nature of most biopsy specimens represent obstacles to accumulating large cohorts of tumors with excess tissue sufficient for molecular analyses, and, as a result, the drivers of SebCA remain incompletely understood [28].

While increased surveillance has led to improved patient survival, therapeutic advancement has not exhibited the same progress, and effective medical interventions for locally invasive, recurrent, and metastatic disease are lacking [29]. Orbital exenteration is ultimately performed in 13–23% of cases to achieve local control, with regional (nodal) or distant metastasis occurring in 8% of cases and disease-associated mortality occurring in 6–10% of cases [1,30,31,32]. The primary factor limiting our understanding of SebCA biology and the development of precision therapies is the lack of reliable models in which to evaluate tumorigenesis and the response to therapeutic intervention. Research on sebaceous malignancies has historically relied upon immortalized human sebocyte cultures with limited success, but, recently, two cell lines derived from human ocular adnexal SebCA were established at another institution and used for in vitro testing [29,33,34,35,36]. Another group has used short-term primary cultures of eyelid SebCA for expression analysis and testing [37]. Here, we describe the molecular and genomic features, cellular behavior, and therapeutic response of three new additional primary ocular adnexal SebCA cell lines with a correlation to the patient tumors from which they were derived. We use these models to begin testing the potential of MYC, and the neoplastic changes it promotes, as a target for therapy in SebCA.

## 2. Results

### 2.1. Clinical and Microscopic Characterization of Sebaceous Carcinoma Specimens

The SebCA tumoral tissues used to generate the JHH-SebCA01, JHH-SebCA02, and JHH-SebCA03 cell lines were derived from patients undergoing the initial surgical resection of their primary tumors. None had chemo- or radiation therapy to the carcinoma prior to this resection. JHH-SebCA01 was derived from a 58-year-old female with a left upper eyelid poorly differentiated SebCA that was surgically resected using frozen sections with clear margins and negative MAP biopsies. Two years after surgery, she was diagnosed with a glioblastoma multiforme and is currently on chemotherapy, immunotherapy, and high-dose steroids. JHH-SebCA02 was derived from a 67-year-old male with a recurrent right upper eyelid SebCA that was resected with clear margins on frozen sections; yet, it ultimately, demonstrated growth within the orbit and metastasis to the lymph nodes within the neck requiring exenteration and neck lymph node dissection. Following radiation and six weeks of cisplatin therapy, there was no evidence of recurrence at the last follow-up, 10 months after surgery. JHH-SebCA03 was derived from a 69-year-old female with a right upper eyelid poorly differentiated SebCA that was resected with clear margins on frozen sections and negative MAP biopsies. At the most recent recheck, 13 months postoperatively, there was no evidence of recurrence. 

**Figure 1:**
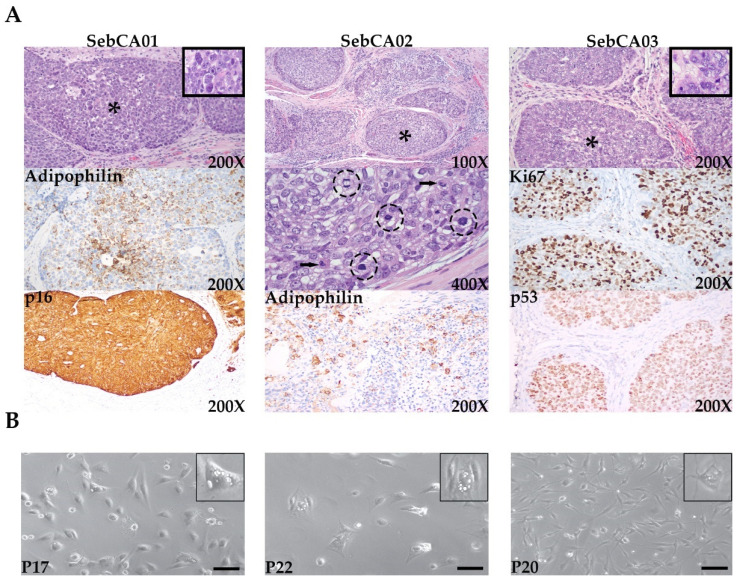
Appearance of surgical specimens and corresponding cultured cells. (**A**) Hematoxylin and eosin-stained sebaceous carcinomas from the three patients showing basaloid nodules of tumor in the eyelid dermis (upper panels, representative nodules marked by asterisks). A higher-magnification view of SebCA02 (second row, middle panel) highlights markedly atypical nuclei and cytoplasmic vacuoles, which were seen in all three tumors, as well as mitotic figures (circles) and apoptotic bodies (arrows). Representative immunohistochemical staining for adipophilin and Ki67 shows sebaceous differentiation and high levels of proliferation. Tumor cells were also positive for p53 and p16, while the surrounding stroma was negative. (**B**) Sebaceous carcinoma cell lines grown in conditional reprogramming cell conditions were adherent and polypoid to spindled with short processes; variable numbers of vacuoles were noted in their cytoplasm (insets). P: passage number. Scale bar: 20 μm.

All three surgical specimens were analyzed microscopically and immunohistochemically. Hematoxylin and eosin staining revealed classical morphological characteristics of SebCA in all three cases (Figure 1A). Large nodules of a somewhat basophilic tumor were present in the dermis, and a subset of larger nodules showed comedo-necrosis. With respect to the intraepithelial extension of the carcinoma, the SebCA03 specimen revealed regions with tumor spread within the palpebral conjunctiva. The peripheral palisading and clefting characteristics of basal cell carcinoma were absent. As shown in the lower-magnification SebCA02 image, stromal inflammation was present around some tumor nodules. At higher magnification, the tumor cells showed large, pleomorphic nuclei and abundant mitotic figures and apoptotic bodies. At least focally in each case, the tumor cell cytoplasm contained small- to medium-sized vacuoles characteristic of sebaceous differentiation. On immunohistochemical stains, adipophilin staining around these vacuoles aided in confirming sebaceous differentiation in all three tumors (Figure 1A) [29]. Additional immunohistochemical stains included Ki67, showing a markedly increased proliferation, and all three cases were positive for p53 and p16 (Figure 1A). High-risk HPV testing was not performed in any of the three cases.

### 2.2. Establishment and Molecular Characterization of Sebaceous Carcinoma Cell Lines

Resection specimens were taken fresh from the operating room to pathology, where they were dissected under sterile conditions, and abnormal tissue, grossly consistent with carcinoma, was placed in media. Cells were then dissociated and serially passaged in conditional reprogramming cell (CRC) media. After a few (2 or 3) passages, all three cell lines began to exhibit a uniform appearance and stable growth characteristics, which were maintained throughout the subsequent passages. All three lines grew in an adherent fashion, with a morphology similar to that of the SebCA cell line BP50 previously described by another group (Figure 1B) [29]. The cells were spindled with short processes, and variable numbers of vacuoles were noted in their cytoplasm (Figure 1B insets). In order to confirm that the established lines came from their corresponding primary tumors, and that no contamination had taken place, short tandem repeat (STR) analysis was performed after 12 passages. As shown in Table 1, the STR profiles of the cell lines all matched those of the patient samples from which they were derived. The highest passage number to date for each cell line is as follows: SebCA01 (passage 24), SebCA02 (passage 25), and SebCA03 (passage 28). At the last passage, the cells were all still growing well in CRC media.

DNA alterations were also characterized in the cell lines using next-generation sequencing (NGS). In SebCA01 and SebCA02, we were able to compare mutations and other changes in the cell lines to those in the surgical specimen from which it was derived. However, for SebCA03, an insufficient amount of the tumor remained after the initial clinical work-up for NGS analysis. In SebCA01, cells’ alterations in the oncogenes *ECTR2L*, *BCORL1*, *BRD4*, and *ERBB4* were shared with the surgical specimen, although the variant allele frequencies (VAF) tended to be higher in the cultured cells (Table 2). In contrast, a *TP53* deletion detected in the primary tumor was not present in the SebCA01 cells, while they contained a *BRCA* alteration not identified in the primary tumor. In SebCA02, *POLD1*, *DNM2*, *EPHA3*, *HISTH1D*, *HISTH2BO*, *RUNX1*, *ZNF703*, and *RAD51D* alterations were also present in the primary tumor, while two *ZFHX4* alterations were only identified in the cell line, and *TP53* and *MKI67* changes present in the primary tumors were not detected in the corresponding cultures. Finally, *CBLB*, *CHD1*, *MAP3K14*, *MST1R*, *SPEN*, and *SYNE1* alterations were identified in the SebCA03 line. Paired, non-neoplastic tissue was not sequenced in the three patients; thus, it is not clear whether the molecular alterations, particularly those with a VAF near 50%, are germline. No copy number variations were identified at any loci evaluated with our NGS panel. 

### 2.3. Characterization of Sebaceous Carcinoma Cell Line Growth and Differentiation

**Figure 2:**
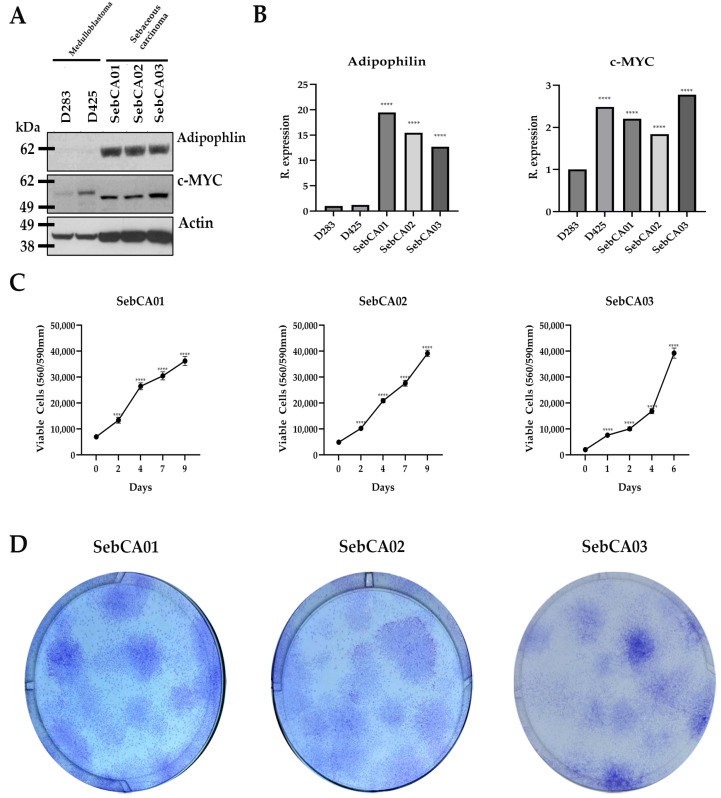
Analysis of sebaceous carcinoma cell growth and differentiation. (**A**) Western blot analysis confirmed strong adipophilin expression in all three sebaceous carcinoma cell lines, while this marker of sebaceous differentiation was not abundant in two established medulloblastoma cell lines (D283 and D425). Sebaceous carcinoma cells had similar MYC protein levels to the medulloblastoma lines, which are known to be dependent on high MYC expression. (**B**) Quantification of adipophilin and MYC protein expression levels (normalized to actin expression). **** *p* ≤ 0.0001. (**C**) All three sebaceous carcinoma cell lines grew robustly when evaluated with Cell-Titer blue assays (**** *p* ≤ 0.0001; *n* = 6 wells per line). (**D**) The three lines were capable of anchorage-dependent colony formation when seeded at single cell density, with images taken after 14 days.

To evaluate the expression of differentiation and stem cell markers in cultured cells, protein extracts were analyzed on Western blots. All three SebCA lines expressed adipophilin protein, which is expressed in association with lipid vacuoles in more differentiated tumor cells, while two established medulloblastoma lines (D283 and D425) lacking sebaceous differentiation were negative (Figure 2A,B). Previous research showed that the *MYC* locus is amplified in a subset of SebCA [27]. Ectopic MYC expression in the skin can also drive the proliferation of adnexal epithelial cells and the enlargement of sebaceous glands [38]. MYC protein expression was, therefore, also assessed in our SebCA cell lines. Assessing the MYC expression in cultured non-neoplastic sebaceous tissue was not possible, and we, therefore, focused on comparing the expression in our SebCA cells to established cancer lines in which MYC was known to be highly expressed and functionally significant. A subset of Group 3 medulloblastoma highly express MYC protein, and two medulloblastoma lines (D283 and D425), reported to show an elevated MYC expression, were used as positive controls [39,40]. 

Our SebCA01, 02, and 03 cells all showed MYC protein expression (after normalization to loading using actin) similar to the expression in D425 medulloblastoma, and approximately twice that in D283 cells (Figure 2A,B). The observed differences in the molecular weight of MYC between the medulloblastoma and SebCA lines is likely a consequence of post-translational modifications and cleavages [41]. 

Next, we examined the overall growth in vitro, as well as the capacity for colony formation of the SebCA lines. All three lines showed robust growth at passage 15 in CRC media, with a doubling time of two to three days, although SebCA03 grew slightly faster than the other two lines (Figure 2C). We also assessed the growth in high serum media, but, while the three lines initially proliferated well under these conditions, over several weeks, they could no longer be passaged. Finally, we examined the clonogenic capacity of the three new SebCA lines. After seeding at a single cell density, anchorage-dependent colonies formed in all three lines, although these were somewhat irregular in shape, and some had a slightly dispersed morphology (Figure 2D). No colonies formed in the anchorage-independent assay using soft agar, and we did not attempt to grow organoids. 

### 2.4. Response of Sebaceous Carcinoma Lines to Chemotherapy

**Figure 3:**
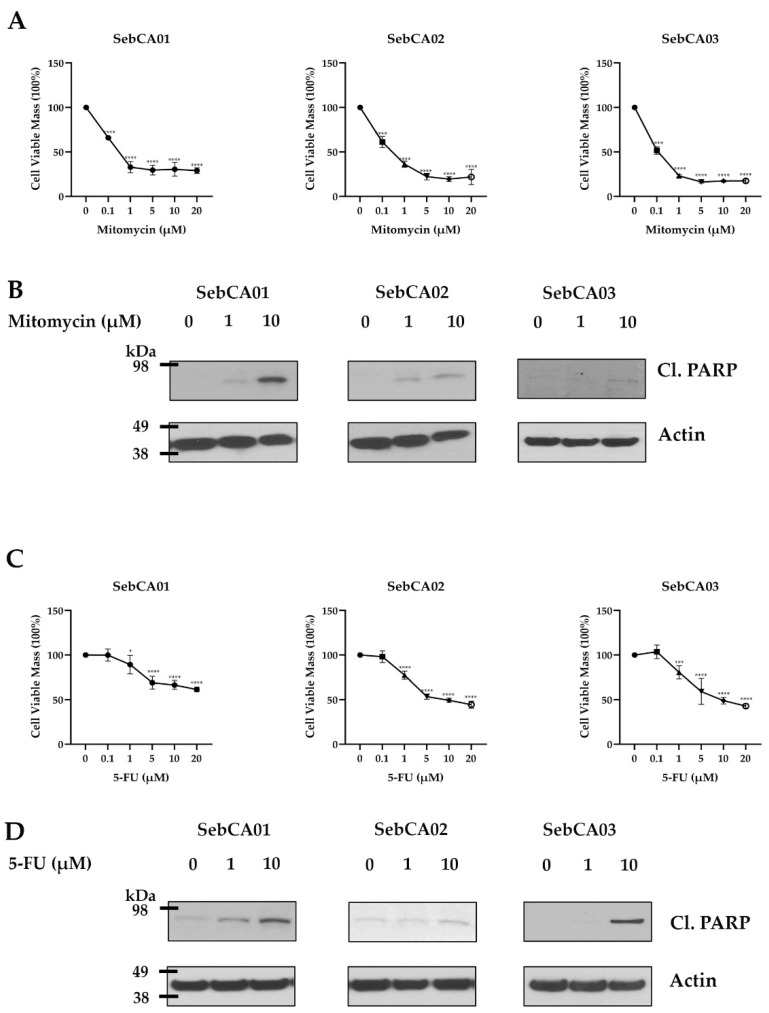
Effects of chemotherapy on sebaceous carcinoma cells. (**A**) Dose-dependent inhibition in viable cell mass growth was seen on MTT assay after 5 days in all three lines with increasing concentrations of mitomycin C (**** *p* ≤ 0.0001, *n* = 6 wells per concentration). (**B**) Induction of cleaved PARP (Cl. PARP) was identified on Western blot after 48 h of treatment, supporting apoptotic effects of the therapy. (**C**,**D**) Growth inhibition (*n* = 6 wells per concentration) and apoptotic induction was also seen following 5-FU treatment. * *p* < 0.05, *** *p* < 0.001, **** *p* ≤ 0.0001.

Mitomycin-C (MMC) and 5-fluorurocil (5-FU) are two chemotherapeutic agents used for the topical treatment of SebCA [36]. Therefore, we investigated whether the growth in viable cells of the three carcinoma lines was inhibited over several days in vitro when these compounds were added to the media. MMC and 5-FU treatments both significantly inhibited cell growth in a dose-dependent manner compared to controls (Figure 3A,C). Mitomycin showed 60% or greater decreases in cell mass growth at concentrations of 1 μM or higher, while 5 μM 5-FU inhibited the growth of SebCA02 and SebCA03 by almost 50%. In SebCA01, 5-FU was slightly less effective, but still significantly slowed growth. The EC50 values for MMC and 5-FU are presented in Table 3.

**Figure 4:**
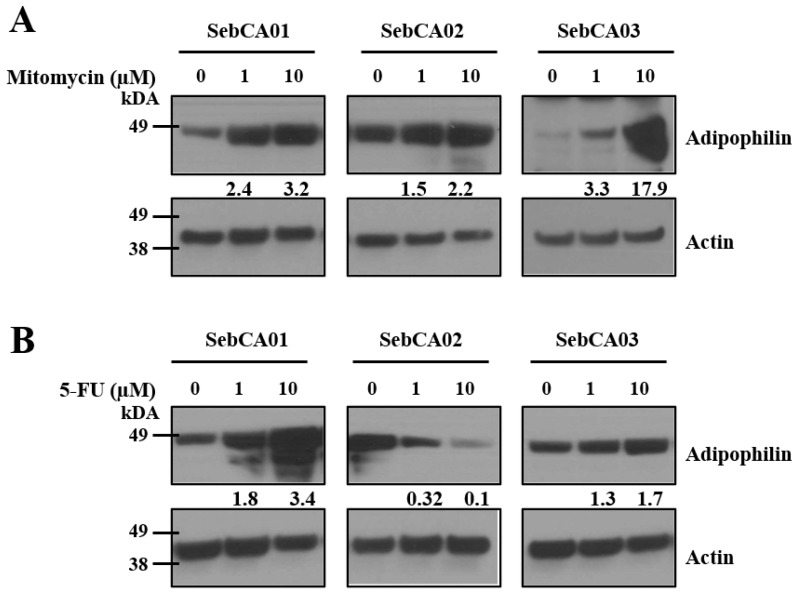
Sebaceous differentiation after therapy. (**A**) Mitomycin-C treatment increased adipophilin protein expression in all three lines, suggesting that it can promote sebaceous differentiation of these carcinoma cells. (**B**) While 5-FU increased adipophilin expression in SebCA01 and SebCA03, a decrease was seen in SebCA02. Numbers between the blots with 1 and 10 μM treatment represent adipophilin expression normalized to actin.

We also examined if the inhibition of growth was at least in part due to the increased apoptotic death of tumor cells by measuring the cleaved PARP protein expression. The increased expression of cleaved PARP was seen in all lines treated with either MMC or 5-FU as compared to controls (Figure 3B,D). Because chemotherapies can target tumor growth by multiple mechanisms, we also measured the adipophilin expression in treated cultures. Marked increases in adipophilin were seen in all three lines after MMC treatment (Figure 4A). In contrast, 5-FU increased adipophilin expression only in SebCA01 and SebCA03, while a decrease was seen in SebCA02 (Figure 4B). Overall, this suggests that both MMC and 5-FU treatment can promote the sebaceous differentiation of these carcinoma cells, leading to the increased expression of adipophilin.

### 2.5. Ongoing Dependence of Sebaceous Carcinoma on Metabolic Changes Associated with MYC

**Figure 5:**
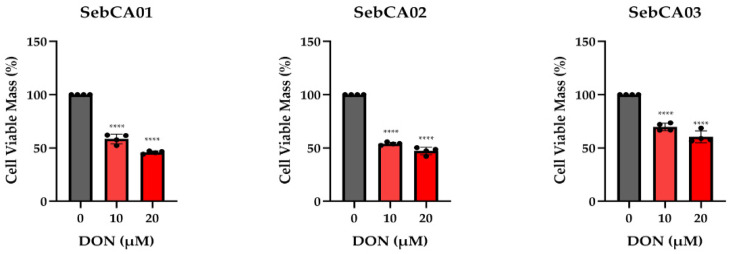
Targeting metabolic changes associated with elevated MYC. Inhibition of glutamine metabolism using DON for 5 days slowed the growth of all three lines in a dose-dependent fashion on MTT assay (**** *p* ≤ 0.0001, *n* = 6 wells per concentration).

Previous research has shown that MYC regulates glutamine metabolism in various types of cancers, and drugs targeting these metabolic alterations have shown clinical potential [42,43]. As shown in Figure 2A,B, all our SebCA cell lines exhibited a high expression of the MYC protein. Consequently, we assessed growth in these cell lines following treatment with the glutamine analog 6-Diazo-5-oxo-L-norleucine (DON), which binds to glutamine active sites on enzymes and selectively inactivates reactions utilizing glutamine. The viable cell mass was significantly inhibited by DON treatment in our three SebCA cell lines in a dose-dependent manner compared to the control (Figure 5).

## 3. Discussion

Sebaceous carcinoma represents a rare, yet aggressive, tumor of the ocular adnexa that is clinically problematic given its propensity to spread intraepithelially and metastasize, leading to highly morbid surgery and, in rare cases, death [44]. Current therapies for ocular adnexal SebCA that can minimize the need for exenteration, including cryotherapy, radiation, and topical chemotherapy, such as MMC and interferon-alpha, show only limited efficacy [8,45]. In an effort to improve therapies for this patient population, we have sought to better understand the molecular drivers of this tumor, focusing on potentially targetable alterations. 

In this study, we were able to establish three cell lines from ocular adnexal SebCA resected at the Wilmer Eye Institute using conditional reprogramming cell techniques, designated JHH-SebCA01, JHH-SebCA02, and JHH-SebCA03. These represent relatively novel cell lines, as only two other institutions have reported the establishment of cell lines for these tumors [29,36,46]. While the three previously reported cell lines were grown in high serum media, in our study, we used specialized “conditional reprogramming” culture techniques with media which are optimized for senescence-prone epithelial cells and tumors, first described by Liu et al. [47]. The conditional reprogramming cell technique has been modified in our laboratory, and used to generate other cell lines including low-grade gliomas of the optic pathways and brain [48]. In brief, this involves adding conditioned media from irradiated fibroblasts as well as a ROCK inhibitor to media lacking serum.

Molecular testing was performed using STR analysis and NGS to confirm that each cell line genetically reflected the tumor from which it was derived. These results show a good correlation overall between genetic alterations in the original tumors and the corresponding cell lines. Interestingly, *TP53* alterations were identified in the tumors giving rise to both SebCA01 (VAF 17.6%) and SebCA02 (VAF 73.6%), but not in the paired cell cultures; thus, based on our NGS data, none of our three lines are *TP53* mutants. This is similar to the findings of Rong et al., where *TP53* alterations were also lost in their cell line as compared to the surgical specimen [29]. They believed that this was likely secondary to intratumoral genetic heterogeneity, and it is possible the *TP53* mutant tumor subclone in the primary tumors was poorly represented in the portions of the surgical specimens used to generate our cell lines. Interestingly, the eyelid SebCA line reported by Gu and colleagues maintained a *TP53* mutation in culture [46]. It is not clear why this group succeeded in maintaining *TP53* alterations in their cells while ours and Rong et al. could not. The Gu and Rong studies both used similar high-serum conditions, making it less likely that serum played an important role in the ability to maintain *TP53* alterations in culture. Either dominant negative constructs or deletion using CRISPR could be used in the future to assess the effects of p53 loss in our SebCA lines.

In terms of how the mutations present in the cell lines correlate with those previously reported in SebCA, several alterations reported here have been documented in tumoral tissue with the NGS and whole-exome sequencing approaches. Alterations at the *BRCA1* and *BRCA2* locus have been previously reported in 2/13 (15%) of ocular adnexal SebCAs evaluated by NGS with the mutations observed here, in the SebCA01 cell line [27]. This same study reported a missense mutation at the *POLD1* locus in 1/13 tumors sequenced, and our current investigation similarly confirmed a *POLD1* alteration in SebCA02 [27]. Mutations affecting members of the ERBB receptor tyrosine kinase family, including *ERBB2*, have been demonstrated in a cohort of SebCA, with an alteration at the *ERBB4* locus occurring in SebCA01 in the current study [49]. A deletion at the *SPEN* locus was observed in SebCA03, and missense mutations at this same locus have been previously observed in 2/29 ocular adnexal SebCA [12]. Interestingly, mutations in *SPEN*, a transcriptional suppressor, have been previously reported in epithelial neoplasms of the breast, colon, ovary, and nasopharynx [50,51,52]. However, the clinical impact of these and the other alterations detected in our SebCA lines has not yet been elucidated, and many are worth studying further in order to determine their functional importance. Further, it is not entirely clear what sort of gain-of-function, loss-of-function, or neomorphic effects the alterations we have identified here will have. Finally, high-risk HPV can play a role in both SebCA and head and neck carcinomas, but the presence of this virus has not been determined in our cells [19,53].

Current topical therapies that are used for ocular adnexal SebCA, including MMC and 5-FU, demonstrated a dose-dependent response in all three cell lines. Two prior studies evaluating the SebCA cell line response to MMC demonstrated similar low micromolar dose-dependent reductions in viability to our three lines; however, 5-FU was effective at a somewhat lower micromolar concentration in our lines as compared to previous reports [36,46]. It is not clear if these differences in sensitivity to 5-FU were due to intrinsic properties of these tumors, or if the CRC media used played a role. Interestingly, adipophilin protein, which we used as a surrogate marker for sebaceous differentiation, showed an increased signal intensity on the Western blot after treatment, suggesting that at least a subset of cells surviving chemotherapy are better differentiated [54]. 

One of the most interesting findings of possible clinical importance from our prior study was the amplification of the *MYC* locus in a subset of cases [27]. Increased MYC activity has been shown in proliferating keratinocytes, and its role in physiologic epithelial differentiation is well-documented [55,56]. Additionally, diffuse MYC immunolabeling has been demonstrated in eyelid SebCA [57]. In this study, we, therefore, further investigated the pathogenic role and ongoing requirement of MYC using our new lines. While we focus here on the role of MYC, other stem cell markers including ALDH1A1, CD44, and CD133 could be examined in the future, along with other signaling pathways such as WNT which have been reported to affect sebaceous gland growth and differentiation [58].

MYC protein has been detected in each of our three cell lines by Western blot, at similar or greater levels as in two established Group 3 medulloblastoma cell lines that are known to be MYC-driven [59,60]. Several groups have shown that high MYC levels can alter glutamine metabolism in cancer, and sensitize tumor cells to therapies targeting glutamine [42,61,62,63]. Therefore, we treated all three cell lines with DON, a glutamine analogue shown to inhibit other MYC-amplified tumors [42]. DON inhibited the growth of all three lines in a dose-dependent fashion, providing support for an ongoing requirement for MYC in SebCA, and suggesting that it represents a potentially important therapeutic target. Additional studies targeting MYC in vivo, either directly or via the suppression of metabolic changes, are warranted. 

In summary, we have generated three new SebCA cell lines, confirmed the ongoing expression of the sebaceous marker adipophilin in vitro, and shown that the cultures are useful for preclinical testing. One limitation of the lines is that they require maintenance in CRC media, as shifting them to high-serum conditions results in slower growth, which progresses to growth arrest. This growth arrest in serum may be due to cellular senescence; however, this was not directly evaluated. A final potential issue is that these cell lines have only been passaged 24 to 28 times, and, while they are still growing robustly at the last passage without signs of senescence, it remains undetermined whether they represent fully immortalized lines. 

## 4. Materials and Methods

### 4.1. Immunohistochemistry of Sebaceous Carcinomas

Immunolabeling of formalin-fixed, paraffin-embedded (FFPE) tumor specimens was performed by the Johns Hopkins Department of Pathology clinical laboratory using 5 μm sections on a Ventana benchmark Ultra platform and ultraView kit reagents (Tucson, AZ, USA). Normal Meibomian gland was used as a positive control for adipophilin staining, while primary antibody was removed in negative controls. Slides were assessed by a board-certified pathologist (C.G.E). 

### 4.2. Cell Line Establishment and Reagents

Three SebCA tumors were obtained from patients after their agreement to participate in research. The study was reviewed and received ethical approval by the Johns Hopkins Medicine Human Research Protection Program, with a consent protocol and form approved by the Institutional Review Board. Informed consent was obtained from all subjects involved in the study.

Fresh tumor was washed in phosphate-buffered saline solution (PBS) three times and minced, and then incubated with collagenase A solution (200 U/mL) in Hank’s balanced salt solution (HBSS) for 3 h. The dissociated tumor cells were centrifuged at 93× *g* for 5 min at room temperature (RT), and then supernatant was removed and cells were transferred to 60 mm cell culture dished and cultured in CRC medium at 37 °C and 5% CO_2_ conditions. The cells were sub-cultured routinely when cell density exceeded 80%. To prepare CRC conditioned medium, 3T3 fibroblast cells were cultured in F medium (25% F-12, 75% DMEM, 10% FBS, 1× GlutaMAX, and 5 µg/mL insulin) for 48–72 h. The cultured media were then collected and filtered. The filtered medium was mixed with F medium (1:1 ratio) containing 25 ng/mL hydrocortisone, 8.4 nmol Cholera toxin, 10 ng/mL human recombinant epidermal growth factor (EGF), and 5 µM ROCK inhibitor for use with SebCA cells. 

Adipophilin antibody (#393A-14) and 5-Fluorouracil were purchased from Millipore Sigma (St. Louis, MO, USA); MYC (c-Myc) antibody (#5605), cleaved PARP antibody (#9541), and mitomycin C were obtained from Cell Signaling Technology (Danvers, MA, USA); and Beta-Actin antibody (SC-4778) was sourced from Santa Cruz Biotechnology (Dallas, TX, USA). 6-Diazo-5-oxo-L-norleucine (DON; #S8620) was acquired from Selleckchem (Houston, TX, USA). The Cell-Titer blue cell viability assay kit was purchased by Promega (Madison, IA, USA), and Thiazolyl Blue Tetrazolium Bromide (MTT) powder was obtained from Millipore Sigma (St. Lois, MO, USA).

### 4.3. Short Tandem Repeat (STR) Analysis

STR analysis of 17 independent genetic sites was conducted by the Genetic Resources Core Facility (Johns Hopkins, Baltimore, MD, USA) using a PowerPlex 18D short tandem repeat DNA analysis kit (Promega Corporation, Madison, WI, USA). Data were analyzed using Genemapper v5.0 software (Applied Biosystems; Waltham, MA, USA). Each PCR setup included both positive and negative controls. Verification of allele designation for each locus within the positive control kit was conducted for accuracy, and the negative water control was ensured to exhibit no amplification products. 

### 4.4. Next-Generation Sequencing (NGS)

Manual microdissection of FFPE tissue sections followed by DNA extraction and next-generation sequencing (NGS) was performed in the Johns Hopkins Molecular Diagnostic Laboratory using standard clinical protocols and the UCSC version hg19 (NCBI build GRCh37) human reference sequence genome assembly. The Solid Tumor Panel used 435 cancer-related genes, and a complete list can be found at https://pathology.jhu.edu/jhml-services/assets/test-directory/SolidTumorPanel-II_GeneList_v9.0.pdf. URL last accessed on 21 September 2024. Sequences were examined for point mutations and small insertion/deletion mutations in all 435 loci, while, for 64 of these, copy number variations (CNVs) were also reported. 

### 4.5. Cell-Titer Blue and MTT Assays

Cells were seeded onto 96-cell plates at a density of 500 cells per well and then incubated for 24 h, with six wells examined per experimental condition. For the evaluation of cell growth, cells were incubated with Cell-Titer blue solution (overnight incubation at 37 °C) on the indicated date. The fluorescence was measured at 560/590 nm for the Cell-Titer-blue-solution-added plates. To assess change in viable cell mass in therapeutic studies, cultures were treated with vehicle or the indicated concentrations of mitomycin, 5-FU, and DON diluted in complete medium for 3–5 days. The products formed by addition of the MTT solutions were dissolved in dimethyl sulfoxide (DMSO) and the absorbance was measured at 570 nm. The data are presented as a percentage of the control group. 

### 4.6. Anchorage-Dependent Colony Formation Assay

Five hundred SebCA cells were seeded onto 6-well plate per well, and then incubated for two weeks until colonies were visualized. Colonies were fixed with 100% methanol for 10 min, and stained with 0.002% crystal violet solutions (Sigma, St. Louis, MO, USA), and washed with deionized water two times. The colonies were imaged and counted using the ImageJ program (v. 1.54k; National Institutes of Health, Bethesda, MD, USA). 

### 4.7. Western Blot Analysis

Established SebCA cells were lysed with RIPA buffer (Sigma, St. Louis, MO, USA) with protease and phosphatase inhibitor cocktails (Roche, Indianapolis, IN, USA). Normalized proteins were subjected to SDS-PAGE and electrically transferred to polyvinylidene difluoride (PVDF) membranes (Bio-Rad, Hercules, CA, USA). Membranes were blocked with 3% BSA in tris-buffered saline (TBS) containing 0.01% of Tween-20 for 1 h. The membranes were incubated with primary antibodies diluted 1:1000 in 3% BSA in TBST overnight at 4 °C with the antibodies c-MYC, adipophilin, cleaved PARP, and β-actin, and then secondary antibodies were incubated at 1:5000 dilution for 1 h at RT. Membranes were washed three times with TBST and visualized by using enhanced chemiluminescence (ECL). 

### 4.8. Statistical Analysis

For all in vitro experiments, the number of replicates is described in Results, and data are displayed as means ± standard deviation (SD). The data were calculated and analyzed with GraphPad Prism (v. 10.1.2; San Diego, CA, USA). Statistical significance of in vitro or in vivo data was determined using a one-way ANOVA, followed by Dunnett’s multiple comparisons test. A *p*-value of less than 0.05 was considered significant.

## 5. Conclusions

These new ocular adnexal SebCA cell lines should prove useful for interrogating the functional role of MYC and other possible molecular drivers, and for preclinical testing in vitro. Ultimately, this will serve to help develop targeted therapy for SebCA to minimize the need for aggressive surgical resection and allow a better treatment for metastatic disease.

## Figures and Tables

**Table 1:** STR analysis of three primary SebCA tumors and corresponding cell lines.

Loci	SebCA01		SebCA02		SebCA03	
	Tumor	Cell Line	Tumor	Cell Line	Tumor	Cell Line
*AMEL*	X	X	X, Y	X, Y	X	X
*CSF1PO*	12, 13	12,13	12	12	10	10
*D13S317*	12	12	8, 11	8, 11	11	11
*D16S539*	10	10	11, 14	11, 14	11, 12	11, 12
*D18S51*	14, 20	14, 20	14, 16	14, 16	13, 16	13, 16
*D19S433*	13, 14	13, 14	13, 14	13, 14	14, 15	14, 15
*D21S11*	30, 31.2	30, 31.2	29, 30	29, 30	28, 30	28, 30
*D2S1338*	20, 24	20, 24	20, 23	20, 23	20, 23	20, 23
*D3S1358*	16	16	15	15	17, 18	17, 18
*D5S818*	11	11	9, 11	9, 11	13	13
*D7S820*	10, 11	10, 11	8, 11	8, 11	8, 13	8, 13
*D8S1179*	14	14	10, 12	10, 12	13, 15	13, 15
*FGA*	19, 23	19, 23	22	22	19	19
*Penta D*	8, 12	8, 12	9	9	11, 12	11, 12
*Penta E*	7	7	11, 23	11, 23	9, 15	9, 15
*TH01*	6, 9.3	6, 9.3	7	7	6, 7	6, 7
*TPOX*	8, 11	8, 11	8, 11	8, 11	8, 11	8, 11
*vWA*	16, 17	16, 17	17	17	17	17

**Table 2:** Mutations in primary sebaceous carcinoma tumors and corresponding cell lines.

Sample	Tumor				Cell Line			
	Gene	Base Change	AA_Change	%VAF	Gene	Base Change	AA_Change	%VAF
SebCA01	*ECT2L*	A>G	p.Q455R	21.53	*ECT2L*	A>G	p.Q455R	46.5
	*BCORL1*	T>C	p.L1460P	28.79	*BCORL1*	T>C	p.L1460P	49.3
	*BRD4*	C>G	p.E1270D	48.08	*BRD4*	C>G	p.E1270D	46.9
	*BRD4*	G>A	p.P1184L	50.23	*BRD4*	G>A	p.P1184L	49.6
	*ERBB4*	T>C	p.I892V	48.52	*ERBB4*	T>C	p.I892V	50.6
	*TP53*	GCTGCACAGGGCAGGT>G	p.T140_Q144del	17.61	*BRCA2*	G>A	p.V917I	17.99
SebCA02	*POLD1*	A>G	p.D893G	47.18	*POLD1*	A>G	p.D893G	47.7
	*DNM2*	CCTT>C	p.F804del	5.83	*DNM2*	CCTT>C	p.F804del	41.2
	*EPHA3*	G>A	p.R136Q	43.82	*EPHA3*	G>A	p.R136Q	45.6
	*HIST1H1D*	A>G	p.V159A	56.39	*HIST1H1D*	A>G	p.V159A	48.3
	*HIST1H2BO*	A>T	p.M1?	35.84	*HIST1H2BO*	A>T	p.M1?	50.3
	*RUNX1*	C>G	p.G69R	36.39	*RUNX1*	C>G	p.G69R	50.2
	*ZNF703*	A>G	p.T178A	54.09	*ZNF703*	A>G	p.T178A	48.9
	*NFKBIZ*			48	*NFKBIZ*			44.8
	*RAD51D*			10	*RAD51D*			43.7
	*TP53*	TGA>T	p.S392Rfs*78	73.62	*ZFHX4*	G>A	p.A1684T	41.41
	*MK167*	C>T	p.A2673T	29.05	*ZFHX4*	C>A	p.P3119T	39.5
SebCA03	Insufficient Sample				*CBLB*	A>G	p.F814S	48.57
					*CHD1*	C>T	p.V96I	45.49
					*MAP3K14*	G>A	p.R849W	46.32
					*MST1R*	G>A	p.P8L	46.12
					*SPEN*	GCCCCCA>G	p.T3246_P3247del	22.3
					*SYNE1*	C>T	p.E7905K	47.36

**Table 3:** EC50 values (mean ± SD) for three primary mitomycin-C (MMC)- and 5-fluoruracil (5-FU)-treated sebaceous carcinoma cell lines.

Cell Line	Mitomycin (μM)	5-FU (μM)
SebCA01	0.12 ± 0.02	1.88 ± 0.95
SebCA02	0.20 ± 0.07	1.29 ± 0.17
SebCA03	0.08 ± 0.01	1.63 ± 0.47

## Data Availability

The original contributions presented in the study are included in the article. Further inquiries can be directed to the corresponding authors.
