# Correction: Lee et al. Establishment and Characterization of Three Human Ocular Adnexal Sebaceous Carcinoma Cell Lines. *Int. J. Mol. Sci.* 2024, *25*, 10183

**DOI:** 10.3390/ijms26189147

**Published:** 2025-09-19

**Authors:** Su-Chan Lee, Cornelia Peterson, Kaixuan Wang, Lujain Alaali, James Eshleman, Nicholas R. Mahoney, Emily Li, Charles G. Eberhart, Ashley A. Campbell

**Affiliations:** 1 Department of Pathology, The Johns Hopkins University School of Medicine, Baltimore, MD 21205, USA; suchanlee80@gmail.com (S.-C.L.);; 2 Department of Comparative Pathobiology, Tufts University Cummings School of Veterinary Medicine, North Grafton, MA 01536, USA; cornelia.peterson@tufts.edu; 3 Department of Oncology, The Johns Hopkins University School of Medicine, Baltimore, MD 21205, USA; 4 Department of Ophthalmology, The Johns Hopkins University School of Medicine, Baltimore, MD 21205, USA

In the original publication [1], there was a mistake in Figure 4B. Specifically, the actin bands for the SebCA02 cell line were inadvertently duplicated from those of the SebCA01 cell line during figure assembly. The original raw western blot data was reviewed, and it was confirmed that the correct actin bands for SebCA02 were available and valid. The corrected Figure 4B panel appears below. The authors state that the scientific conclusions of the paper are unaffected. This correction was approved by the Academic Editor. The original publication has also been updated.

## Figures and Tables

**Figure 4:**
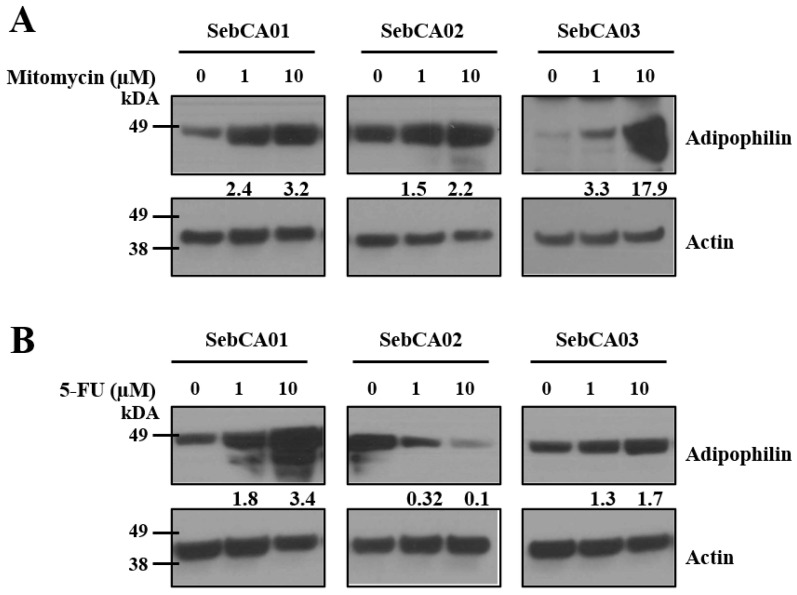
Sebaceous differentiation after therapy. (**A**) Mitomycin-C treatment increased adipophilin protein expression in all three lines, suggesting that it can promote sebaceous differentiation of these carcinoma cells. (**B**) While 5-FU increased adipophilin expression in SebCA01 and SebCA03, a decrease was seen in SebCA02. Numbers between the blots with 1 and 10 μM treatment represent adipophilin expression normalized to actin.
